# Online video games and patient–staff power relations. A qualitative study of care and custody in forensic psychiatry

**DOI:** 10.1111/jpm.12813

**Published:** 2022-01-07

**Authors:** Morten Deleuran Terkildsen, Harry G Kennedy, Christian Jentz, Lisbeth Uhrskov Sørensen

**Affiliations:** ^1^ DEFACTUM ‐ Public Health & Health Services Research Aarhus Central Denmark Region Denmark; ^2^ Department of Forensic Psychiatry Aarhus University Hospital Psychiatry Denmark; ^3^ Trinity College Dublin University of Dublin Ireland; ^4^ Central Mental Hospital Dundrum Ireland; ^5^ Department of Clinical Medicine Faculty of Health Aarhus University Denmark

**Keywords:** forensic, nursing role, psychosocial intervention, recovery

## Abstract

**What is known on the subject?:**

- Frontline forensic mental health staff often face challenges when providing recovery‐orientated care, as they must balance between caring for the forensic psychiatric patient and at the same time ensuring safety and security for all other patients and staff at the ward.
- Research shows that balancing between care and custody in everyday clinical practice is possible, but more practical nursing studies showing ways of balancing power relations are needed to guide clinical practice.
- Online video games are increasingly recognized as promising new tools to promote social relations, establish competencies and re‐articulate power relations in therapeutic environments.

**What the paper adds to existing knowledge?:**

- This paper provides insights into how using online video gaming interventions may influence the establishment of social power relations of staff and forensic psychiatric patients. It adds to existing research by providing a conceptual way to study and understand how mental health nurses may balance between care and custody, delivering care to accommodate patients' needs without compromising safety and security at the ward.
- This study answers a call in current research by providing qualified knowledge regarding the use of online video gaming to build and sustain therapeutic relations in mental health care.

**What are the implications for practice?:**

- Our paper suggests that balancing between care and custody is possible by using online video gaming interventions in forensic psychiatry. It moreover provides practice‐close knowledge that may inspire and guide clinical mental health nurses to further develop online video gaming interventions in mental health care for the benefit of their patients.

**Abstract:**

**Introduction**

In recovery‐oriented care, forensic psychiatric nurses must engage in care relationships with forensic psychiatric patients (FPs) whilst focussing on ward security. Online video games (OVGs) may provide a platform for negotiating power and social relations. Studies showing how OVG interventions may influence power balances in forensic psychiatric care are needed to guide clinical practice.

**Aim**

Our aim was to study how power relations were articulated between FPs and staff in an OVG intervention in a Danish forensic psychiatric ward.

**Method**

Data consist of three months of observational data and interviews with three staff members and six patients. We used sociologist Pierre Bourdieu's framework of field, power and capital to analyze data.

**Results**

The OVG intervention consisted of two power fields, “in‐game” and “over‐game.” In‐game concerned the practice of gaming. Over‐game described the organization of the gaming intervention. Specific logics, skills and symbolic capitals drove power in each field.

**Discussion**

Power in‐game was open to FPs and staff, leading to symmetric power relations. Power over‐game was open to staff only, resulting in asymmetrical power relations.

**Implications for practice**

Online video game interventions may facilitate power balancing in forensic psychiatry. These insights may guide the development of new OVG interventions for patients and nurses in mental health care.

## 1 INTRODUCTION

Research is increasingly paying attention to the dilemmas of providing high‐quality and secure recovery care within forensic psychiatry (Mann et al., 2014; Marshall & Adams, 2018; Martin & Street, 2003). Recovery‐orientated care emphasizes equality in relations and relies on therapeutic relationships where power is more symmetrically distributed between nurses and forensic psychiatric patients (FPs) (Mann et al., 2014). Care and treatment based on more symmetric power relations are challenging in forensic psychiatry. FPs are double stigmatized by being both vulnerable and in need of care and treatment but also dangerous and in need of risk management (Brooker & Ullmann, 2008). Consequently, nurses need to engage with FPs as therapeutic and custodial agents (Adshead, 2000; Livingston et al., 2012). As custodial agents, nurses manage potential risks FPs pose to themselves, other FPs, staff and society in general (Gildberg et al., 2010; Maden, 2007; Martin & Street, 2003). This custodial role may promote asymmetrical power in patient–nurse relationships, placing power with the nurses. Also, the custodial role is considered counterproductive to the therapeutic nursing role and recovery‐oriented care (Cashin et al., 2010; Mann et al., 2014; Marshall & Adams, 2018; Martin & Street, 2003; Slade, 2009) However, research argues that care and custody may successfully coexist in forensic psychiatry (Peternelj‐Taylor & Johnson, 1996).

### 1.1 Rationale

Research increasingly recognizes online video games (OVGs) as a valuable therapeutic intervention in mental health care (Boldi & Rapp, 2021; Rosegrant, 2012; Steadman et al., 2014). When played in a structured form, OVGs may teach participants important individual and social competencies to establish and maintain new social identities and relations. OVGs may also promote feelings of empowerment and respect among participants via group recognition in practice realized via gaming skills (Boldi & Rapp, 2021; Freeman & Wohn, 2017; Granic et al., 2014; Wright et al., 2002). OVGs are also a scene for negotiating power relations (Consalvo, 2007; Toft‐Nielsen & Krogager, 2015). Despite showing promise as a therapeutic instrument in mental health care, few have studied the use of OVG interventions in forensic psychiatric settings. Research states that we need to examine new practices that provide FPs with competencies to strengthen their social relations and provide alternate identities, something often found severely challenged among FPs in forensic psychiatric wards (Barnao & Ward, 2015; Clarke et al., 2016). Furthermore, research must focus on practices where power relations defined by the care‐and‐custody dichotomy in forensic psychiatry are balanced (Dorkins & Adshead, 2011; Hillbrand et al., 2010; Mann et al., 2014; Marshall & Adams, 2018; Martin & Street, 2003; Simpson & Penney, 2011).

### 1.2 Objective

The objective of this qualitative study is to understand how power relations are negotiated between FPs and staff in an OVG intervention in a medium secure forensic psychiatric ward.

### 1.3 Aim

Our paper aims to use the knowledge acquired to inform existing discussions concerning care and custody and provision of secure recovery to guide future mental health nursing practices in forensic psychiatry.

## 2 METHOD

We used a qualitative ethnographic approach with an interpretive analysis to study the relations between FPs and staff in the OVG intervention (Braun & Clarke, 2006; Hammersley & Atkinson, 1995). This social constructivist approach underlines the contextual nature of knowledge and knowledge production and the importance of reflexivity. We therefore viewed OVG as a social practice where participants negotiate meanings within local contexts (Boellstorff, 2006).

### 2.1 Research team and reflexivity

The research team included one anthropologist (AN), two psychiatrists and one mental health nurse. AN collected data from interviews, informal conversations and observations. All researchers participated in the development of the study and analysis. Three researchers had clinical experience with forensic psychiatry. Their expertise was continuously subjected to reflection to prevent potential preconceptions guiding the study.

We informed FPs that the research team had no authority at the ward and could not influence treatment plans. Staff and FPs were told that we would maintain confidentiality. Care was taken to spend equal amounts of time with FPs and staff.

### 2.2 Context

The study was conducted at a Danish medium secure forensic psychiatric ward. The ward has 16 male FPs aged 18–55 years. The ward has the equivalent of 43 full‐time employees and discharges 3–5 FPs per year.

In the intervention, FPs and staff play an OVG (Counter‐Strike GO) together one day weekly from 3.30–9 pm to counter FP isolation and promote social skills. FPs and staff chose the game in unison. In Counter‐Strike GO, two teams of five players compete to either eliminate the opposing team or solve predefined mission goals. The first team to win 16 rounds wins the match (Weblink 1). FPs may ask staff for referral, or other staff may refer the FP. Upon referral, the staff screen FPs by interview and by gaming together. Once accepted into the group, the staff and the FP agree on individual goals (Table 1). Staff evaluate the goals after each session (Table 2). Before admission to OVG, FPs receive a written set of rules for the session (Table 3).

**TABLE 1:** Individually negotiated goals for patients prior to and during the online video game recovery‐oriented intervention in a Danish medium security forensic psychiatric ward

Goal to improve	Examples
Communication	Patients who do not speak very much because of their negative mental health symptoms will be trained on how to communicate when gaming.
	Patients acting as in‐game leaders are trained to guide the group by precise instructions, give kindly worded instructions and avoid abusive verbal outbursts.
Social skills	Patients who are assigned a tedious task in the game are trained to adhere to the mission for the sake of the group.
	Patients who quit in the middle of a game due to, e.g., frustration are asked to stay because it will comprise the chance of winning for the entire team if they quit before the end of the game.
	Good players are instructed to teach less talented players.
Handling aggression	Patients are trained to use positive expressions to praise and recognize their fellow players instead of yelling and being abusive.

**TABLE 2:** Typical schedule for a session of online video gaming in a Danish medium security forensic psychiatric ward

03.30–04.00 pm	Review the program for the session
04.00–04.15 pm	Break
04.15–04.30 pm	Continue review
04.30–05.30 pm	Gaming session with the game of the day
05.30–06.00 pm	Evaluation of the game
06.00–06.45 pm	Dinner and smoking break. The instructors eat together to evaluate the session
06.45–09.00 pm	New game based on the number of participants
09.00–09.30 pm	Instructors document the session for each patient in the electronic patient file

**TABLE 3:** Written information for patients participating in online video gaming intervention in a Danish medium security forensic psychiatric ward

The online video gaming group is a social activity seeking to provide a fun and rewarding community mixed with healthy competition and good team spirit.
The group is a structured treatment that aims to strengthen your social competencies and tolerance towards other people. The group is an arena for relationship‐building and training social skills. The social focus precedes the competitive aspect at all times. The instructors will regularly offer training sessions connected with the regular weekly meetings where participants are expected to attend.
**How to join the group:** Ask the online video gaming staff or your contact person.
**When:** Wednesdays 3.30–09.00 pm.
**Attendance:** You are expected to attend training sessions from 3.30 pm.
**Cancellation:** Everyone can have a bad day or an important appointment, but for the team's sake, report your cancellation as soon as possible to an instructor or your contact person.
**Team spirit:** Good team spirit precedes good team performance. We expect you to try to avoid discussions about guilt and mistakes. We expect you to play constructively with curiosity to improve your skills. We expect you to be loyal to the team and the in‐game leader's decisions. Give a high five rather than complain.
**Breaks:** Between games, we encourage you to leave the room and take a 5‐min break in the garden to relax and get ready for the next round.
**Hygiene:** Indoor climate is vital for concentration and performance when gaming for longer periods. The gaming room has several hot PCs, and players may sweat with great concentration. Therefore, it is essential to attend the gaming session in clean clothes and that you shower during the day. Please avoid coming to the gaming room straight from physical exercise.
**Cleaning up:** After the session, please clean your space and wipe all surfaces with a disinfectant swap, including your headset. Tidy up your headset, your chair and clean up any rubbish.
**Food and drink:** Eating is not allowed in the gaming room. This includes snacks, e.g. chips, peanuts and popcorn.
We look forward to playing with you. The staff

The staff instructors are certified OVG coaches. Each OVG session begins with a teaching and training module (Table 2). Afterwards, the group plans the remaining session and chooses the in‐game leader, either a FP or staff.

### 2.3 Participant recruitment and selection

This study is based on dialogues and interviews with six FPs and three staff members in the gaming group at the time of the study. The group consisted of seven FPs (19–54 years) and three staff members.

The staff responsible for organizing the intervention consisted of one educator and two nurse assistants (31–35 years), all with a minimum of 3 years of work experience in forensic psychiatry.

All participants provided informed consent to participate in the study. Their consent included allowing observation, participating in interviews and recording informal conversations. One FP declined the interview.

### 2.4 Ethics

We followed the American Anthropological Association's guidelines for qualitative research (www.ethics.americananthro.org). We obtained informed signed consent before the study—start based on oral and written information. It was repeated orally before every interview that participants could always withdraw their consent. The ward consultant considered all participating FPs able to give informed consent. AN removed names and identifiable details to anonymize the results. All FPs were offered to read the final draft to ensure they felt unidentifiable. In Denmark, studies that do not include human biological material and are based on questionnaires and interviews do not require permission from the Ethical Committee according to the Promulgation of the Law on Ethical management of Health Science Research Projects (Weblink 2).We made an inquiry to the hospital ethical committee. They confirmed that according to Danish law, the study did not need approval from an ethical board.

### 2.5 Data collection and processing

During 3 months in 2019, AN collected data by participant observation at the ward and during OVG (Boellstorff, 2012; Spradley, 1980). Data also included informal dialogues and interviews to provide thick ethnographic descriptions (Hastrup, 2003; Wolcott, 2005). During fieldwork, AN participated in 13 hours of observation and engagement in informal dialogues at the ward from 8 am to 3 pm to understand life at the ward. From 3.30–9 pm, AN observed the OVG intervention for a total of 60 h, where he noted dialogue between participants and their physical actions in the gaming room. AN also participated in OVG to understand gaming practices (Boellstorff, 2012). Informal dialogues and observations were written down. Initially, we focussed upon observing and discussing with FPs and staff how they experienced life at the ward, their relations and the OVG intervention. We observed who did what, when and for what reasons, and who was perceived as in charge during gaming. After observations, we discussed with FPs and staff what being in charge meant for them and their relations. Based on these data, AN developed interview guides, in which the topics of ward life and gaming, gaming competencies and roles, and roles, relations and power were further explored. The interview guide was developed based on discussions with the research team during fieldwork. Interviews took place at the ward, lasted 15–60 min, and were transcribed verbatim. We coded data with Nvivo 12.

### 2.6 Data analysis

We followed Braun and Clarke's (2006) method to analyze data. It involves multiple readings of data whilst gradually developing and validating the analysis. The analysis process covers the data collection phase. Our analysis followed Braun & Clarke's six steps.

1. Our analysis began during fieldwork. AN would meet with the research group at regular intervals to discuss observations and ideas to explore. After fieldwork, AN first conducted a thorough reading of field notes and interviews focussing on how the participants articulated social relations, understandings of the OVG intervention and how they distributed power. This reading supported the research aim as well as a preliminary understanding achieved during fieldwork. The content was discussed with the research group to identify analytical ideas further.
2. AN reflexively reread field notes and interviews to develop initial codes. Codes were discussed with the research group. Subsequently, AN coded the material.
3. Codes were structured into potential themes. Though not initially theory‐driven, we found a solid theoretical resonance between these codes, potential themes and the framework of Pierre Bourdieu. It became the subject of further exploration (Section 2.7).
4. An additional code set, based on Bourdieu's framework, was developed and discussed with the group. The material was recoded by AN, structured into potential themes and subthemes and then reviewed in the group.
5. Themes were defined and named. Two central themes and several subthemes emerged.
6. The remainder of the analysis was performed by AN and regularly discussed with the research group.

We used method triangulation during data collection and researcher triangulation during the analysis to develop the results. AN discussed initial findings and concepts with FPs and staff during the fieldwork to further qualify the results. During the final analysis, AN visited the ward to discuss the results with FPs and staff.

### 2.7 Theory

We followed the theoretical framework of sociologist Pierre Bourdieu to analyze the relationship between power, context and gaming practice. We approached the intervention as a practice unfolding within local *fields* (Consalvo, 2007; Toft‐Nielsen & Krogager, 2015). According to Bourdieu, fields are understood as social spaces (Bourdieu, 1990, 1998). These are specialized domains of practice, centred around a field logic referring to self‐evident and therefore nonreflected values, rules and interests of that particular field (Andersen et al., 2015). An individual's position within a field is defined by his/her access to different forms of capital acting in accordance with the logic of that field (Bourdieu, 1998; Bourdieu & Wacquant, 1992). Capital may be understood as encompassing economic, social and cultural as well as critical symbolic forms. Symbolic capital refers to recognition and prestige (Bourdieu, 1998). To obtain recognition, one has to possess the kind of capital perceived as important in a field. Possession of symbolic capital and power is highly connected, and power is abstracted from relations between people, according to their accumulated symbolic capital within a field (Andersen et al., 2015; Bourdieu, 1990, 1998).

## 3 RESULTS

The observations and interviews with staff and FPs regarding the OVG intervention revealed how power between FPs and staff could be understood as highly contextual and delineated between two power fields: “in‐game” and “over‐game.”

### 3.1 Power “in‐game”

We suggest that power in‐game is tied to the practice of gaming together. This form of power emerged from a logic viewing good gaming as the result of a collective enterprise. Power in‐game, among FPs and staff, was perceived as distributed according to the possibility of obtaining and the possession of specific individual and social gaming skills. Following the game's logic, these skills were considered essential and provided group recognition to those possessing them.

### 3.2 Winning as a collective enterprise

Both staff and FPs explicitly stated that OVG's central logic was to win a match against another team. One FP explained:

Winning! I like to win I mean, that's what it's all about after all. (Dialogue field note 11)

Moreover, they expressed that winning should be achieved by a successful team effort. The idea that winning should be accomplished by a team was formulated in the intervention's official ruleset. FPs and staff who had been gaming before entering the intervention mentioned that this was an inherent logic of team gaming in the broader gaming community. A staff member uttered:

It's when all sort of pitch in. I mean when there is some sort of togetherness in the game. We have been goddam together on this (…) something we have done as a team. (Interview ST)

Correspondingly, one FP elaborated how winning via team effort was more important than individual glory:

If I die thirty times without shooting anyone, but we still win (as a team), then it's really a nice match for me. (Interview FP)

Staff and FPs also expressed that specific skills were needed and valued in this logic of winning as a collective enterprise. These were social gaming skills and individual gaming skills.

### 3.3 Social gaming skills in‐game

Staff and FPs framed social gaming skills as specific practical skills that were necessary to learn in order to be able to perform as a team player.

One FP expressed, “*It's about working together*.”

Another FP elaborated how this involved *accepting and sticking to the role the team gave you*, e.g. playing defensively for the sake of the team even when an offensive push could offer personal wins. Team communication would equally be framed as an essential social gaming skill. One staff member said “team communication is everything.” A FP elaborated how the ability to communicate to team members was the hallmark of a good player and a good game:

That you are good at communicating (to your teammates) and observing stuff (for the team) (…) good tone and good communication. (Interview FP)

Both FPs and staff acknowledged that obtaining these skills was essential for successful participation in‐game. Neglecting to obtain them could ruin the entire team, as it would undermine the game's logic. One staff member recalled a particular player lacking these skills:

That's why it was sometimes frustrating to play with (FP name) because he disobeyed the rules (i.e., He didn't play for the team). (Interview ST)

### 3.4 Individual gaming skills in‐game

Apart from exhibiting good social skills, it was equally important to possess individual gaming skills to be successful.

Being skilled at hitting the enemy (aim) in‐game was perceived as an important individual skill by both staff and FPs. One FP said:

It's important that you're good at aiming. Him (FP name), him (FP name) and him (staff name) are probably best. They're important and can really make a difference for the team. I'm coming along, but their aim is good. He (FP name) can pull off some crazy headshots. (Dialogue field note 6)

Correspondingly, a FP talked about one of the staff member's superior ability to hit the enemy:

I especially like to play with him (staff name) on the team because I like to win. (Interview FP)

Another valued and important individual skill was “gaming sense” or “understanding the game.” This included knowing when to push areas of the map, utilize the equipment in the game, and position oneself when engaging the enemy. One FP said:

Knowing these things is what makes you a good player and what sets you apart from others. (Dialogue field note 10)

This shared logic of winning as a collective enterprise and realizing it through individual and social gaming skills created new positions for FPs and staff.

### 3.5 Skills and power positions in‐game

Both parties used “gamers,” “players,” or “gaming nerds” to describe themselves and teammates in‐game. These descriptors were seen as diminishing differences between staff and FPs. One staff member stated:

When I'm in here (the gaming room), we are all just gaming nerds. (Interview ST)

A FP used similar wording in an interview, calling all participants players:

You don't consider that they're staff (and we are FPs). In there, we just all play together. (Interview FP)

When the FPs talked about life at the ward in general, they called the staff members “staff.” Power positions in‐game were expressed as highly dependent on the participants' social and individual skills. One staff member described it this way:

When we are in there, it's the skills in the game that decide who you are, and what you can do. (Dialogue field note 12)

For staff and FPs alike, the possibilities for acquiring skills and becoming a vital resource for the team were seen as tied to the individual player's diligence in playing the game, following the rules, and learning the ropes. Both groups perceived they had equal possibilities in obtaining these skills.

Those acknowledged by all participants as having already obtained these skills were perceived as particularly vital to the success of the team, exemplified by a FP:

He (FP name) is really one of the good ones. He can hit, and he knows tactics and when to say things. Therefore, he is very important to the team if we want to win. (Dialogue field note 9)

Revealingly, differences in social and individual gaming skills carved out a hierarchy in‐game. Both staff and FPs saw high degrees of social and individual gaming skills defining who could be “leaders of the team” in‐game. Leaders had the right to dictate orders to fellow players, regardless of being FP or staff. One FP uttered:

In there, patients also get to decide. He (FP name) is good, and he (staff name) and (staff name) also listen when he gives orders. (Dialogue field note 7)

One staff member complemented the statement:

We know that (FP name) and (FP name) are team captains because they have played it so much. (Interview ST)

Overall, the mutual social acceptance of a gaming hierarchy based on gaming skills as a form of capital led to re‐articulations of power in‐game. All saw this as contrasting the established FP–staff relations outside the OVG intervention.

### 3.6 Power “over‐game”

In the analysis, we interpreted power over‐game to refer to the intervention's professional clinical and organizational part. Power over‐game emerged from the staff's perception that the gaming intervention could be a potent forensic psychiatric treatment if it followed specific organizational and gaming rules. Power over‐game was distributed according to the possession of a particular professional form of knowledge, based on a clinically relevant education.

### 3.7 OVG as a professional treatment: Clinicians and power over‐game

All staff members described the OVG intervention as a treatment option that could potentially teach FPs important social skills to control aggression, increase their concentration and break their frequent periods of isolation. They also described it as a way to monitor FPs' behaviour and psychiatric symptoms and observe their ability to handle stress. One staff member mentioned:

It's a treatment group, not a cozy leisure group. We have many assignments (for the FP). There is teaching and they have goals set up, each of them. (Interview ST)

The staff emphasized that the intervention was regulated by explicit rules stapled to the gaming room door (Table 3). FPs could only enter the group by referral. Admittance was cleared with staff. The staff evaluated each FP after the gaming session and documented a summary in the patient record. FPs who disobeyed the rules were excluded. Once a FP had been allowed to partake, participation was mandatory. Absence was only allowed with a valid excuse. What constituted a valid reason was decided by the staff. Before gaming sessions, the staff would often seek out FP to ensure participation.

The knowledge, skills and safety responsibilities required to organize the OVG intervention as treatment (over‐game) were seen as open to staff only. The staff viewed the ability to produce and enforce the OVG intervention's organizational rules as a professional clinical task for which they possessed specific professional knowledge, abilities and responsibility for therapeutic safety. One staff member said:

It's one thing to be good at gaming, but we have a professional angle here. We can see what can work and what can't, differently than the patient can […]. We need to look at the safety issue for the other patients and ourselves […]. It can't all be open to debate because we need to fit this intervention not only with the rest of the department but also with the patients' disease. (Dialogue field note 13)

The staff were explicit and articulate concerning the distinction between in‐game and over‐game competencies.

I can give him (FP name) responsibility for the tactics and what we do in the game, but all the stuff around the game (…) how we speak to each other and such, it is still necessary that I am there. (Interview ST)

Thus, whilst power in‐game could be symmetrically distributed between FPs and staff, power over‐game was recognized as positioned with the staff. A staff member explained that more symmetric distribution of power in‐game did not translate to power over the gaming room:

We are not equal (in the sense of power in the room: Danish) but equal (in the gaming sense: Danish). (Interview ST)

Forensic psychiatric patients were very aware of the rules and the organization of the intervention and recognized the unique clinical position that gave to the staff the right to produce and enforce these rules. FPs were invited to put forward suggestions for developing the intervention. Yet, despite making suggestions to improve the intervention (e.g. suggest alternative schedules), FPs verbally acknowledged that the power to make a final decision rested with the staff due to their position as clinical professionals. One FP said:

We still know that they're staff, and you have to do as they say in there. They(staff) have rules (for the room) like no saying fuck and cursing, no hitting the keyboard. There are rules you have to follow. (Interview FP)

Another FP emphasized this distinction as self‐evident:

In this place, the staff set the agenda (for the intervention). Of course, that's how it is. They say something, and you do as you are told. (Interview FP)

Despite acknowledging the staff's power over‐game, this was not a significant issue and was rarely mentioned in FP interviews.

## 4 DISCUSSION

We found that the OVG intervention consisted of two power fields: “in‐game” and “over‐game.” In‐game concerned the practice of gaming and over‐game the organization of the intervention. Power in‐game was equally open to FPs and staff, leading to symmetric power relations. By contrast, power over‐game was open to staff only, leading to asymmetrical power relations (Table 4).

**TABLE 4:** Definition and elements of “Power In‐game” vs. “Power Over‐game” in online video game sessions in a medium secure forensic psychiatric ward, Denmark, 2019

	In‐game	Over‐game
Definition	The practice of gaming together	The organization of a professional treatment intervention
Logic	Winning as a social enterprise	Providing therapeutic safety and security effective clinical treatment
Symbolic capital	Individual and social gaming skills	Clinically relevant education
Patient–staff power distribution	Symmetric	Asymmetric

Forensic psychiatric patients need treatment to relieve suffering and reduce the risk of future violence. Forensic psychiatric services' challenge is finding ways to facilitate a process of increasing stability, providing insight, building working alliance and reducing risk (Kennedy, 2002). Even though this is a complex task, finding ways of balancing between care and custody in practice is vital in forensic nursing care (Dorkins & Adshead, 2011; Hillbrand et al., 2010; Mann et al., 2014; Marshall & Adams, 2018; Martin & Street, 2003; Simpson & Penney, 2011).

Our study of a new OVG intervention demonstrated that power between FPs and staff was a nuanced and field‐bound notion. Working for a common cause and realizing it via cooperation is essential to many OVGs (Frostling‐Henningsson, 2009). By following Bourdieu's concepts of field and power (Andersen et al., 2015; Bourdieu, 1990, 1998; Bourdieu & Wacquant, 1992), our study demonstrated that when staff and FPs played OVG together, they developed a common logic in which winning as a collective enterprise should be accomplished as team efforts using social and individual gaming skills. As found in our study, following such explicit gaming rules and appropriating specific individual and social skills for the team's benefit have been underlined by several video game studies as inherent components of successful OVG play (Freeman & Wohn, 2017; Kim et al., 2017; Seo & Jung, 2016).

In mental health care interventions, these game mechanisms may help stimulate prosocial behaviour among patient groups where social functioning is challenged (Boldi & Rapp, 2021; Granic et al., 2014). Significantly, they may also help promote stronger therapeutic relations by redefining staff–patient social roles and offering a sense of sharing meaningfulness on equal terms (Rosegrant, 2012). Following Rosegrant (2012), we found that gaming together re‐articulated existing staff–FP categories replacing them with "players". This promoted mutual respect and recognition of roles and abilities outside the roles of FP and staff. Establishing such relationships through OVG may forefront FPs' competencies, sense of own worth, personal resources and a sense of responsibility (Coffey, 2006; Mezey et al., 2010; Rask & Brunt, 2007; Resnick et al., 2005). They can also help overcome alienation and isolation by allowing a sense of belonging to a group, referenced as “self‐transcendence” (Wharewera‐Mika et al., 2020). Moreover, by favouring a common identity of being players, the often highly stigmatized FP identity (Brooker & Ullmann, 2008) became downplayed during gaming.

It may seem counterproductive to play a violent first‐person shooter video game with FPs in forensic psychiatric wards. Yet, research indicates no current links between playing OVG and committing real‐world violent acts (Granic et al., 2014). This resonates with our experience, as staff and consultants did not observe any increase in aggression after the FPs and staff started playing OVG.

Gaming skills, roles and hierarchical power are highly intertwined (Consalvo, 2007; Freeman & Wohn, 2017; Toft‐Nielsen & Krogager, 2015). This makes OVG interventions relevant when aiming to progress from power asymmetry towards power symmetry. Recognizing social and individual skills as the defining symbolic capital when in‐game, FPs and staff viewed their possibilities for acquiring this symbolic capital as equally open to both. This situation promoted a sense of power equality in‐game between them. It also provided new possibilities for repositioning power from staff to FPs in‐game.

By contrast, the organizational issue of the OVG intervention (over‐game) was defined by a clinical logic. This logic resulted in power relations closely resembling the asymmetric power relations between FPs and staff described as inherent to forensic psychiatry (Caplan, 1993; Mann et al., 2014; Martin & Street, 2003; Rask & Brunt, 2007). Defining the OVG intervention as a psychiatric treatment, the staff articulated a familiar position, where knowledge and skill are necessary to make rules for participation in a treatment intervention came from formalized professional education (see (Andersen et al., 2015; Helman, 1990)).

Equal relationships are essential for recovery (Slade, 2009). Psychiatric nurses' interests and approaches may be steered by care ideas based on relational equality and power symmetry with FPs (Martin & Street, 2003). Yet, custodial orientations may easily prevail skewing power relations towards asymmetry between them (Maden, 2007). Our paper illustrates that when organized as an intervention, the unique features of OVG may provide a platform where the balancing of power is possible on different levels.

Interestingly, this distribution of power to FPs need not be an all‐or‐nothing task for positive FP–nurse relationships to emerge. In our study, the staff engaged in redistributing power to patients in‐game whilst retaining control over the intervention (over‐game). Distinguishing between “power in‐game” and “power over‐game” may offer ways to understand the therapeutic value of OVG in secure forensic hospitals. At the same time, it is essential to recognize the protective value of professional boundaries when maintaining relational security (Royal College of Psychiatrists, 2015), particularly in therapeutically safe environments where patients prone to violence can be challenged concerning sensitive but dysfunctional beliefs and emotions (Tighe & Gudjonsson, 2012).

Online video game is a growing phenomenon and for many children an inherent part of social life (Granic et al., 2014). In 2020, counterstrike GO had more than 1.3 million online players (Weblink 3). Therefore, using OVG in future mental health care could provide staff with a common platform to establish meaningful relations with mental health care patients for which OVG is a part of life. In forensic psychiatry, OVG interventions could help promote alternative identities, recognize FP competencies, destigmatize and ultimately help balance power in FP–nurse relations. Yet, we need to understand balancing by taking a microperspective on OVG, acknowledging that power as a notion is related to concrete field practices.

## 5 LIMITATIONS

In Bourdieu's framework, fields are sites of stability and change with ongoing power struggles between established participants and newcomers to the field (Bourdieu & Pierre, 1977; Bourdieu, 1998). The FPs and staff had already gamed together for an extended period before our data collection. No new FPs entered during our study. Furthermore, the FPs had already progressed from an admission ward to a rehabilitative medium secure forensic psychiatric ward due to prior progress in reducing the risk of violence (Müller‐Isberner et al., 2007) and building working alliance and trust (Donnelly et al., 2011). Consequently, the introduction of new participants in the group during the study period could have instigated power struggles influencing the logic, symbolic capitals and power positions presented in our analysis.

This qualitative study explores the social relations and issues of power following an organized therapeutic OVG intervention. Yet, casual OVG has been shown to promote similar therapeutic effects. Whether the therapeutic effects related to the in‐game field could be obtained by staff and FPs simply gaming as a leisure social activity should be investigated further. Further quantitative studies are needed to explore the possible effect on outcomes such as gaming disorder, social interaction and ward atmosphere.

## 6 CONCLUSION

Our results suggest that nurses can balance care and custody by using OVG interventions in forensic psychiatry.

## 7 RELEVANCE STATEMENT

Research is lacking concerning how forensic psychiatric nurses may balance between engaging in care relationships with patients and focussing on ward security. This paper addresses this research gap. Qualitatively, it explores how participation in a video‐gaming intervention for forensic psychiatric staff and patients influences the balance between the different power relations of the care‐and‐custody dichotomy. It conceptualizes how balancing between care and custody for nurses may be understood and achieved using online video gaming. The insights may help develop future recovery‐orientated interventions using video games for nursing staff.

## CONFLICT OF INTEREST

The authors declare no conflicts of interests related to the manuscript.

## AUTHOR CONTRIBUTIONS

Anthropologist Morten Deleuran Terkildsen (MD) designed the study. Medical Doctor Lisbeth Uhrskov Sørensen (LU), Nurse Christian Jentz (CJ) and Medical Doctor Harry Kennedy (HK) revised the study protocol. MD collected and performed data analyses and drafted the manuscript. LU, CJ and HK provided crucial intellectual input on study design and contributed substantially to interpreting findings and manuscript revision.

## ETHICS STATEMENT

The study, involving interviews with forensic psychiatric patients at a medium secure ward in Denmark, was entered and registered at the Legal Office at Central Region, Denmark (file number:1‐16‐02‐289‐19). According to the Consolidation Act on Research Ethics Review of Health Research Projects, Consolidation Act number 1338 of 1 September 2020, section 14(2) notification of observation and interview projects to the research ethics committee system is only required if the project involves human biological material. Therefore, this study may be conducted without an approval from the ethics committees.

## Supporting information

Supplementary Material

## Data Availability

Research data are not shared due to privacy or ethical restrictions.

## References

[jpm12813-bib-0001] Adshead, G. (2000). Care or custody? Ethical dilemmas in forensic psychiatry. Journal of Medical Ethics, 26(5), 302–304. 10.1136/jme.26.5.302 11055029 PMC1733273

[jpm12813-bib-0002] Andersen, R. S. , Tørring, M. L. , & Vedsted, P. (2015). Global health care‐seeking discourses facing local clinical realities: Exploring the case of cancer. Medical Anthropology Quarterly, 29(2), 237–255. 10.1111/maq.12148 25355457

[jpm12813-bib-0003] Barnao, M. , & Ward, T. (2015). Sailing uncharted seas without a compass: A review of interventions in forensic mental health. Aggression and Violent Behavior, 22, 77–86. 10.1016/j.avb.2015.04.009

[jpm12813-bib-0004] Boellstorff, T. (2006). A ludicrous discipline? Ethnography and game studies. Games and Culture, 1(1), 29–35. 10.1177/1555412005281620

[jpm12813-bib-0005] Boellstorff, T. (2012). Ethnography and virtual worlds: A handbook of method. Princeton University Press.

[jpm12813-bib-0006] Boldi, A. , & Rapp, A. (2021). Commercial video games as a resource for mental health: A systematic literature review. Behaviour & Information Technology, 1–37. 10.1080/0144929X.2021.1943524

[jpm12813-bib-0007] Bourdieu, P. (1977). Outline of a theory of practice. Cambridge University Press.

[jpm12813-bib-0008] Bourdieu, P. (1990). The logic of practice (R. Nice Trans.). Polity Press.

[jpm12813-bib-0009] Bourdieu, P. (1998). Practical reason. Polity Press.

[jpm12813-bib-0010] Bourdieu, P. , & Wacquant, L. J. D. (1992). An invitation to reflexive sociology. Polity Press.

[jpm12813-bib-0011] Braun, V. , & Clarke, V. (2006). Using thematic analysis in psychology. Qualitative Research in Psychology, 3(2), 77–101. 10.1191/1478088706qp063oa

[jpm12813-bib-0012] Brooker, C. , & Ullmann, B. (2008). Out of sight, out of mind. The state of mental healthcare in prison. Policy Exchange.

[jpm12813-bib-0013] Caplan, C. A. (1993). Nursing staff and patient perceptions of the ward atmosphere in a maximum security forensic hospital. Archives of Psychiatric Nursing, 7(1), 23–29. 10.1016/0883-9417(93)90019-S 8476314

[jpm12813-bib-0014] Cashin, A. , Newman, C. , Eason, M. , Thorpe, A. , & O'Discoll, C. (2010). An ethnographic study of forensic nursing culture in an Australian prison hospital. Journal of Psychiatric and Mental Health Nursing, 17(1), 39–45. 10.1111/j.1365-2850.2009.01476.x 20100305

[jpm12813-bib-0015] Clarke, C. , Lumbard, D. , Sambrook, S. , & Kerr, K. (2016). What does recovery mean to a forensic mental health patient? A systematic review and narrative synthesis of the qualitative literature. The Journal of Forensic Psychiatry & Psychology, 27(1), 38–54. 10.1080/14789949.2015.1102311

[jpm12813-bib-0016] Coffey, M. (2006). Researching service user views in forensic mental health: A literature review. The Journal of Forensic Psychiatry & Psychology, 17(1), 73–107. 10.1080/14789940500431544

[jpm12813-bib-0017] Consalvo, M. (2007). Cheating: Gaining advantage in videogames. MIT Press.

[jpm12813-bib-0018] Donnelly, V. , Lynch, A. , Devlin, C. , Naughten, L. , Gibbons, O. , Mohan, D. , & Kennedy, H. G. (2011). Therapeutic alliance in forensic mental health: Coercion, consent and recovery. Irish Journal of Psychological Medicine, 28(1), 21–28. 10.1017/S0790966700011861 30199989

[jpm12813-bib-0019] Dorkins, E. , & Adshead, G. (2011). Working with offenders: Challenges to the recovery agenda. Advances in Psychiatric Treatment, 17(3), 178–187. 10.1192/apt.bp.109.007179

[jpm12813-bib-0020] Freeman, G. , & Wohn, D. Y. (2017). Social support in eSports: Building emotional and esteem support from instrumental support interactions in a highly competitive environment. Paper presented at the CHI PLAY 2017 – Proceedings of the Annual Symposium on Computer‐Human Interaction in Play. Association for Computing Machinery, Inc. 435–447.

[jpm12813-bib-0021] Frostling‐Henningsson, M. (2009). First‐Person shooter games as a way of connecting to people: “Brothers in Blood”. CyberPsychology & Behavior, 12(5), 557–562. 10.1089/cpb.2008.0345 19817566

[jpm12813-bib-0022] Gildberg, F. A. , Elverdam, B. , & Hounsgaard, L. (2010). Forensic psychiatric nursing: A literature review and thematic analysis of staff–patient interaction. Journal of Psychiatric and Mental Health Nursing, 17(4), 359–368. 10.1111/j.1365-2850.2009.01533.x 20529188

[jpm12813-bib-0023] Granic, I. , Lobel, A. , & Engels, R. C. (2014). The benefits of playing video games. The American Psychologist, 69(1), 66–78. 10.1037/a0034857 24295515

[jpm12813-bib-0024] Hammersley, M. , & Atkinson, P. (1995). Ethnography: Principles in practice (2nd ed.). Routledge.

[jpm12813-bib-0025] Hastrup, K. (2003). Introduktion. Den antropologiske videnskab. In K. Hastrup (Ed.), Into the world. An introduction to anthropological method [Ind I verden. En Grundbog i Antropologisk Analyse] (pp. 9–34). Hans Reitzels Forlag.

[jpm12813-bib-0026] Helman, C. G. (1990). Culture, health and illness. Wright.

[jpm12813-bib-0027] Hillbrand, M. , Young, J. L. , & Griffith, E. E. H. (2010). Managing risk and recovery: Redefining miscibility of oil and water. The Journal of the American Academy of Psychiatry and the Law, 38(4), 452–456. 21156903

[jpm12813-bib-0028] Kennedy, H. (2002). Therapeutic uses of security: Mapping forensic mental health services by stratifying risk. Advances in Psychiatric Treatment, 8, 433–443. 10.1192/apt.8.6.433

[jpm12813-bib-0029] Kim, Y. J. , Engel, D. , Lin, J. Y. , McArthur, N. , Malone, T. W. , & Woolley, A. W. (2017). What makes a strong team? using collective intelligence to predict team performance in league of legends. Paper presented at the Proceedings of the 2017 ACM on the Conference of Computer Supported Cooperative Work and Social Computing, 2316–2329.

[jpm12813-bib-0030] Livingston, J. D. , Nijdam‐Jones, A. , & Brink, J. (2012). A tale of two cultures: Examining patient‐centered care in a forensic mental health hospital. The Journal of Forensic Psychiatry & Psychology, 23(3), 345–360. 10.1080/14789949.2012.668214 22815648 PMC3396355

[jpm12813-bib-0031] Maden, T. (2007). Treating violence: A guide to risk management in mental health. Oxford University Press.

[jpm12813-bib-0032] Mann, B. , Matias, E. , & Allen, J. (2014). Recovery in forensic services: Facing the challenge. Advances in Psychiatric Treatment, 20(2), 125–131. 10.1192/apt.bp.113.011403

[jpm12813-bib-0033] Marshall, L. A. , & Adams, E. A. (2018). Building from the ground up: Exploring forensic mental health staff's relationships with patients. The Journal of Forensic Psychiatry & Psychology, 29(5), 744–761. 10.1080/14789949.2018.1508486

[jpm12813-bib-0034] Martin, T. , & Street, A. F. (2003). Exploring evidence of the therapeutic relationship in forensic psychiatric nursing. Journal of Psychiatric and Mental Health Nursing, 10(5), 543–551. 10.1046/j.1365-2850.2003.00656.x 12956633

[jpm12813-bib-0035] Mezey, G. C. , Kavuma, M. , Turton, P. , Demetriou, A. , & Wright, C. (2010). Perceptions, experiences and meanings of recovery in forensic psychiatric patients. The Journal of Forensic Psychiatry & Psychology, 21(5), 683–696. 10.1080/14789949.2010.489953

[jpm12813-bib-0036] Müller‐Isberner, R. , Webster, C. D. , & Gretenkord, L. (2007). Measuring progress in hospital order treatment: Relationship between levels of security and C and R scores of the hcr‐20. International Journal of Forensic Mental Health, 6(2), 113–121. 10.1080/14999013.2007.10471256

[jpm12813-bib-0037] Peternelj‐Taylor, C. A. , & Johnson, R. (1996). Custody and caring: Clinical placement of student: Nurses in a forensic setting. Perspectives in Psychiatric Care, 32(4), 23–29. 10.1111/j.1744-6163.1996.tb00519.x 9121864

[jpm12813-bib-0038] Rask, M. , & Brunt, D. (2007). Verbal and social interactions in the nurse–patient relationship in forensic psychiatric nursing care: A model and its philosophical and theoretical foundation. Nursing Inquiry, 14(2), 169–176. 10.1111/j.1440-1800.2007.00364.x 17518829

[jpm12813-bib-0039] Resnick, S. G. , Fontana, A. , Lehman, A. F. , & Rosenheck, R. A. (2005). An empirical conceptualization of the recovery orientation. Schizophrenia Research, 75(1), 119–128. 10.1016/j.schres.2004.05.009 15820330

[jpm12813-bib-0040] Rosegrant, J. (2012). Technologically altered reality inside the therapist's office. Psychoanalytic Psychology, 29(2), 226–240. 10.1037/a0025329

[jpm12813-bib-0041] Royal College of Psychiatrists . (2015). Your guide to relational security: See, think, act. NHS.

[jpm12813-bib-0042] Seo, Y. , & Jung, S. (2016). Beyond solitary play in computer games: The social practices of eSports. SAGE Publications. 10.1177/1469540514553711

[jpm12813-bib-0043] Simpson, A. I. F. , & Penney, S. R. (2011). The recovery paradigm in forensic mental health services. Criminal Behaviour and Mental Health, 21(5), 299–306. 10.1002/cbm.823 22113784

[jpm12813-bib-0044] Slade, M. (2009). Personal recovery and mental illness: A guide for mental health professionals. Cambridge University Press.

[jpm12813-bib-0045] Spradley, J. P. (1980). Participant observation. Wadsworth.

[jpm12813-bib-0046] Steadman, J. , Boska, C. , Lee, C. , Lim, X. , & Nichols, N. (2014). Using popular commercial video games in therapy with children and adolescents. Journal of Technology in Human Services, 32, 201–219. 10.1080/15228835.2014.930680

[jpm12813-bib-0047] Tighe, J. , & Gudjonsson, G. H. (2012). See, think, act scale: Preliminary development and validation of a measure of relational security in medium‐ and low‐secure units. Journal of Forensic Psychiatry & Psychology, 23, 1–16. 10.1080/14789949.2012.671336

[jpm12813-bib-0048] Toft‐Nielsen, C. , & Krogager, S. G. S. (2015). Gaming practices in everyday life. An analytical operationalization of field theory by means of practice theory. MedieKultur: Journal of Media and Communication Research, 31, 68–84. 10.7146/mediekultur.v31i58.18741

[jpm12813-bib-0049] Weblink 1 . Rules for counter‐strike GO. Retrieved from https://counterstrike.fandom.com/wiki/Competitive#Differences_from_casual_modes

[jpm12813-bib-0050] Weblink 2 . Danish law on ethical management of health science research projects. Retrieved from https://www.retsinformation.dk/eli/lta/2017/1083

[jpm12813-bib-0051] Weblink 3 . CS:GO player count tops dota 2's all‐time record for the first time ever. Retrieved from https://www.pcgamesn.com/counter‐strike‐global‐offensive/csgo‐player‐count‐record

[jpm12813-bib-0052] Wharewera‐Mika, J. , Cooper, E. , Wiki, N. , Prentice, K. , Field, T. , Cavney, J. , Kaire, D. , & McKenna, B. (2020). The appropriateness of Dundrum‐3 and Dundrum‐4 for Māori in forensic mental health services in New Zealand: Participatory action research. BMC Psychiatry, 20(1), 61. 10.1186/s12888-020-2468-x 32046679 PMC7014731

[jpm12813-bib-0054] Wolcott, H. F. (2005). The art of fieldwork. Altamira Press.

[jpm12813-bib-0055] Wright, T. , Boria, E. , & Breidenbach, P. (2002). Creative player actions in FPS online video games: Playing counter‐strike. Game Studies, 2(2), 103–123.

