# Supplementary material for: Online video games and patient–staff power relations. A qualitative study of care and custody in forensic psychiatry
Source: J Psychiatr Ment Health Nurs. 2022 Jan 7;29(3):493–503. doi: 10.1111/jpm.12813 (PMC9306509; doi:10.1111/jpm.12813)
Supplement: Supplementary file 1 — Supplementary Material [file JPM-29-493-s001.docx]

**Accessible summary**

**What is known on the subject?**

- Frontline forensic mental health staff often face challenges when providing recovery‐orientated care, as they must balance between caring for the forensic psychiatric patient and at the same time ensuring safety and security for all other patients and staff at the ward.
- Research shows that balancing between care and custody in everyday clinical practice is possible, but more practical nursing studies showing ways of balancing power relations are needed to guide clinical practice.
- Online video games are increasingly recognized as promising new tools to promote social relations, establish competencies and re‐articulate power relations in therapeutic environments.

**What the paper adds to existing knowledge?**

- This paper provides insights into how using online video gaming interventions may influence the establishment of social power relations of staff and forensic psychiatric patients. It adds to existing research by providing a conceptual way to study and understand how mental health nurses may balance between care and custody, delivering care to accommodate patients' needs without compromising safety and security at the ward.
- This study answers a call in current research by providing qualified knowledge regarding the use of online video gaming to build and sustain therapeutic relations in mental health care.

**What are the implications for practice?**

- Our paper suggests that balancing between care and custody is possible by using online video gaming interventions in forensic psychiatry. It moreover provides practice-close knowledge that may inspire and guide clinical mental health nurses to further develop online video gaming interventions in mental health care for the benefit of their patients.
